# Editorial Comment: Embryological Development and Topographic Anatomy of Pelvic Compartments-Surgical Relevance for Pelvic Lymphonodectomy

**DOI:** 10.1590/S1677-5538.IBJU.2022.03.04

**Published:** 2022-04-14

**Authors:** Natasha T. Logsdon, Luciano A. Favorito

**Affiliations:** 1 Universidade do Estado do Rio de Janeiro Unidade de Pesquisa Urogenital Rio de Janeiro RJ Brasil Unidade de Pesquisa Urogenital - Universidade do Estado do Rio de Janeiro - Uerj, Rio de Janeiro, RJ, Brasil

Andreas Bayer ^1^, Tillmann Heinze ^1^, Ibrahim Alkatout ^2^, Daniar Osmonov ^3^, Sigmar Stelzner ^4^, Thilo Wedel ^1^

Eur J Radiol. 2021 Sep;142:109885

## COMMENT

The removal of pelvic lymph nodes is a important point during the radical prostatectomy and radical cystectomy. The extension of lymph node dissection is controversial and associated with more complications in post-operative period (1). In this interesting paper the topographic anatomy of pelvic compartments in relation to pelvic lymphonodectomy for rectal, uterine, and prostate cancer are reviewed. The paper has amazing figures showing the anatomy of the pelvic lymph nodes including a interesting description of the lymph nodes of Marcille fossa. The authors concluded that the comprehensive knowledge of pelvic anatomy, the exchange of surgical concepts between specialties, and minimally invasive techniques will optimize pelvic lymphonodectomy and reduce complications.
